# Editorial: Animal Models in Psychiatry: Translating Animal Behavior to an Improved Understanding and Treatment of Psychiatric Disorders

**DOI:** 10.3389/fpsyt.2022.876155

**Published:** 2022-03-24

**Authors:** Neil M. Richtand, Brian H. Harvey, Kurt Leroy Hoffman

**Affiliations:** ^1^Department of Psychiatry, University of California, San Diego, San Diego, CA, United States; ^2^Center of Excellence for Pharmaceutical Sciences, North-West University, Potchefstroom, South Africa; ^3^Center for Research in Animal Reproduction (CIRA), Autonomous University of Tlaxcala-CINVESTAV, Tlax, Mexico

**Keywords:** depression, anxiety, genetics, model organisms, obsessive-compulsive disorder, anorexia nervosa, binge eating

Psychiatric conditions result from interactions between multitudes of risk genes, interacting with developmental environment. While there has been much progress identifying vulnerability genes, and environmental influences, identification of psychiatric biomarkers with utility to advance directed treatments and improve outcome has lagged. To date, neurobiological advances have not translated into improved patient care. Many of these issues may be directly related to the lack of robust translational models to enable pre-clinical research to properly address the fundamental pathology of these illnesses, and so to provide new and improved treatments. Clearly, new strategies and novel paradigms for pre-clinical modeling of psychiatric disorders are needed. The present Frontiers in Psychiatry specialty section on Molecular Psychiatry showcases some recent developments in the field of pre-clinical research in psychiatry that have tackled questions ranging from sex differences in psychiatric illness and genetic and neurodevelopmental risk factors, to strategies for improving the translatability of pre-clinical work to the clinic.

Animal models utilizing behavioral measures of relevance to psychiatric conditions provide potential to identify novel hypotheses of etiology, diagnostic biomarkers, targets for treatment, and predictors of prognosis. Lovick and Zangrossi review the complex nature of sex effects in animal models of anxiety. Their review is particularly pertinent given the preponderance of anxiety and fear studies in male rodents, in contrast to the elevated prevalence of anxiety disorders in female patients. Their findings highlight the need for validation of standardized behavioral models of anxiety in female rodents in order to advance care of both endogenous, as well as substance-induced anxiety conditions.

A contrasting approach to advancing mood disorder research focusing on depression, was taken by Sall et al. They employed an innovative analysis of risk genes for major depression in simple model organisms. Their analysis identified a high degree of interconnection between major depression risk genes, with extensive cross-species conservation of shared biological functions. Their findings suggest molecular pathways with likelihood for involvement in the etiology of major depression, suggesting potential new approaches to prevention and treatment.

Major depression has its origins in genetic predisposition and early life environmental adversity, amongst others. Dual-hit models that combine these elements may provide insight into disease progression and treatment response. In their study targeting major depression, Mncube et al. examined both genetic and environmental contributions to rat behavioral models of relevance to treatment resistant depression. Utilizing the Flinders Sensitive Line rat as an animal model of major depression genetic risk, the authors characterized interactions between genetic risk, social isolation, and antidepressant treatment on behavioral and biochemical outcome measures of relevance to treatment resistant depression. They observed combined effects of genetic vulnerability in Flinders Sensitive Line rats, interacting with social isolation, resulting in behavioral and biochemical measures more resistant to antidepressant treatment. These findings suggest potential for a combined genetic/environmental model with face, predictive and construct validity for treatment-resistant depression.

Obsessive-compulsive disorders—including obsessive compulsive disorder (OCD), hoarding disorder, body dysmorphic disorder, trichotillomania, and skin picking disorder—share the characteristics of repetitive behaviors and/or obsessive, intrusive thoughts. Obsessive-compulsive (OC) traits demonstrate considerable heritability, indicating that genetic factors play an important role in their pathogenesis. Although it is not feasible to assess obsessive-like mental experiences in a non-human animal, it is possible to observe repetitive and compulsive-like behaviors—occurring either spontaneously or induced by some experimental manipulation. Mitra and Bult-Ito review studies of a mouse line that was generated by their group through bidirectional selection of individuals that spontaneously displayed excessively high (“compulsive-like”) or low nest building behavior. Studies of these lines have revealed novel potential neurochemical targets for OCD pharmacotherapy. Moreover, this group reports important effects of gonadal hormones and the oxytocinergic system on compulsive-like behavior in female mice, providing clues on the physiological underpinnings of OC symptoms associated with the female reproductive cycle. These mouse lines present an excellent opportunity to investigate how interactions between genetic liability, physiological state, and environmental factors contribute to the pathogenesis of obsessive-compulsive symptoms.

Anorexia nervosa (AN) is a potentially fatal illness presenting with suboptimal psychological and behavioral therapies, while pharmacological treatments are lacking in therapeutic efficacy. Zhang and Dulawa review the utility of animal models for studying AN, highlighting how little basic research has contributed to a better understanding of the biological mechanisms of the disorder. AN has strong metabolic and psychiatric origins, suggesting its reconceptualization as a metabo-psychiatric disorder. The authors posit that, despite limitations such as an inability to mimic certain psychological constructs of the disorder, the activity-based anorexia (ABA) paradigm allows the reconceptualization of AN in this manner. In so doing the authors highlight how modern circuit-dissecting neuroscience techniques may be invaluable in identifying metabo-psychiatric mechanisms that regulate ABA, as well as genetic variants and gene pathways in AN. This will aid in identifying novel targets and treatment strategies for AN.

Hildebrandt and Ahmari describe a “component assessment” strategy for evaluating rodent models of binge eating (BE, a core symptom of many eating disorders), and ultimately for improving the translation of information gained from these models to the clinic. In this review, they separately consider each of the core components of BE that can be quantitatively measured in the rodent (quantity of food, duration spent feeding, and loss of control over feeding), and then discuss existing rodent models of BE within the context of these outcome measures. This approach, whereby animal models are evaluated based on the relevance of controlled experimental variables and outcome measures to the human condition being modeled, will improve the translatability of pre-clinical work on BE as well as guide the design and development of new animal models for other psychiatric disorders.

As illustrated by the present articles, important controlled experimental variables for preclinical research on psychiatric disorders include sex (Lovick and Zangrossi; Mitra and Bult-Ito) and genetic background and environment (Mncube et al.). The importance of a third variable—the animal species studied—is illustrated by the work of Sall et al. (discussed above) and Santana-Coelho et al. This latter group demonstrates that primate species are advantageous for modeling psychiatric disorders, since their behavior and physiology more closely resemble that of humans. They report the neurodevelopmental effects of prenatal maternal immune activation (MIA, a risk factor for autism and schizophrenia in humans) in marmosets. By administering a battery of tests that assess social behavior and vocal communication, they found that marmosets exposed to prenatal MIA exhibited discrete developmental deficits in both sociality and vocal communication. The results of this study underscore the importance of a comparative approach in animal modeling.

While behaviors—such as fear and “despair” responses, collection of nest material, consumption of palatable food, vocal communication—serve as key outcome measures in animal modeling, it is necessary to have a means to measure neurophysiological correlates of altered behavior, and translate these findings to the clinic. Francoeur et al. describe a low-cost methodology for constructing electrophysiological probes that can be used to register single unit and local field potential activity in behaving rats. Electrophysiological measures taken in rodent models can be related to data obtained from clinical studies involving electroencephalography, functional magnetic resonance imaging, and other measures of brain activity. The methodology described by these authors should facilitate the incorporation of electrophysiological studies to existing rodent models.

In combination, the manuscripts presented in this Research Topic highlight encouraging advances in animal model research. These new directions hold promise for advances in patient care.

## Author Contributions

BH, KH, and NR reviewed and summarized the contributions of the articles described in this Editorial and edited the final submitted manuscript. All authors contributed to the article and approved the submitted version.

## Funding

BH is supported by the South African Medical Research Council.

## Conflict of Interest

The authors declare that the research was conducted in the absence of any commercial or financial relationships that could be construed as a potential conflict of interest.

## Publisher's Note

All claims expressed in this article are solely those of the authors and do not necessarily represent those of their affiliated organizations, or those of the publisher, the editors and the reviewers. Any product that may be evaluated in this article, or claim that may be made by its manufacturer, is not guaranteed or endorsed by the publisher.

